# Phenotypic and genomic characteristics of clinical IMP-producing *Klebsiella* spp. Isolates in China

**DOI:** 10.1038/s43856-024-00439-5

**Published:** 2024-02-21

**Authors:** Congcong Liu, Ning Dong, Yanyan Zhang, Qiaoling Sun, Yonglu Huang, Chang Cai, Gongxiang Chen, Rong Zhang

**Affiliations:** 1 Department of Clinical Laboratory, Second Affiliated Hospital of Zhejiang University, School of Medicine, Hangzhou, China; 2 Department of Medical Microbiology, School of Biology and Basic Medical Science, Medical College of Soochow University, Suzhou, Jiangsu China; 3 College of Animal Science and Technology, College of Veterinary Medicine, Zhejiang Agriculture and Forestry University, Hangzhou, China

**Keywords:** Infectious-disease epidemiology, Antimicrobial resistance

## Abstract

**Background:**

IMP-producing *Klebsiella* spp. (IMPKsp) strains have spread globally, including in China. Currently, the prevalence and genomic characterization of IMPKsp is largely unknown nationwide. Here we aimed to provide a general overview of the phenotypic and genomic characteristics of IMPKsp strains.

**Methods:**

61 IMPKsp strains were obtained from 13 provinces in China during 2016-2021. All strains were tested for their susceptibility to antimicrobial agents by the microdilution broth method and sequenced with Illumina next-generation sequencing. We performed conjugation experiments on thirteen representative strains which were also sequenced by Oxford nanopore sequencing technology to characterize *bla*_IMP_-encoding plasmids.

**Results:**

We find that all IMPKsp strains display multidrug-resistant (MDR) phenotypes. All strains belong to 27 different STs. ST307 emerges as a principal IMP-producing sublineage. *bla*_IMP-4_ is found to be the major isoform, followed by *bla*_IMP-38_. Seven incompatibility types of *bla*_IMP_-encoding plasmids are identified, including IncHI5 (32/61, 52.5%), IncN-IncR (10/61, 16.4%), IncFIB(K)-HI1B (7/61, 11.5%), IncN (5/61, 8.2%), IncN-IncFII (2/61, 3.3%), IncFII (1/61, 1.6%) and IncP (1/61, 1.6%). The strains carrying IncHI5 and IncN plasmids belong to diverse ST types, indicating that these two plasmids may play an important role in the transmission of *bla*_IMP_ genes among *Klebsiella* spp. strains.

**Conclusions:**

Our results highlight that multi-clonal transmission, multiple genetic environments and plasmid types play a major role in the dissemination process of *bla*_IMP_ genes among *Klebsiella* spp. IncHI5 type plasmids have the potential to be the main vectors mediating the spread of the *bla*_IMP_ genes in *Klebsiella* spp.

## Plain language summary

Antibiotic resistance occurs when bacteria evolve to withstand antibiotic drugs. We are aware that a bacteria called *Klebsiella* is rapidly becoming resistant to carbapenems, a class of broad-spectrum antibiotics. In this study, we conducted a genetic and microbiological surveillance study across 13 provinces of China to understand factors that contribute to the growing bacterial drug resistance. We find that the way the multiple bacterial types interact with each other and swap certain genetic material may be the main cause of growing resistance. These findings call for close monitoring of genetic evolution as a matter of public health management strategy.

## Abstract (web summary)

Liu et al. analyze phenotypic and genomic characteristics of clinical IMP-producing *Klebsiella* spp. Isolates in China. Multi-clonal transmission, multiple genetic environments and differing plasmid types all play a role in disseminating *bla*IMP genes among *Klebsiella* spp.

## Introduction

*Klebsiella* spp. are Gram-negative bacteria that can cause opportunistic infections and are a leading cause of community-acquired and nosocomial infections^1^. The emergence and rapid spread of carbapenem-resistant *Klebsiella* spp. (CRKsp), with high morbidity and mortality rates, represents a major public health threat worldwide^2–4^. Production of carbapenemases is the main mechanism contributing to carbapenem resistance in Enterobacteriaceae^5^. The class B metallo-β-lactamases (MBLs) Imipenemases (IMPs) are one of the most important carbapenemases and can hydrolyze almost all β-lactams, such as cephalosporins, and carbapenems, but not the monobactams (i.e., aztreonam)^2^. The first IMP-1 carbapenemase was discovered in *Pseudomonas aeruginosa* in Japan in 1988, and subsequently, IMPs have been detected in *Acinetobacter* spp. and members of the Enterobacteriaceae family^5–7^. Outbreaks caused by strains harboring *bla*_IMP_ genes have been reported in Japan, Australia and China^5–8^.At the time of writing, at least 96 isoforms of IMP carbapenemases have been identified (https://www.ncbi.nlm.nih.gov/pathogens/refgene/#IMP). IMP-encoding genes along with other resistance genes are often located within class 1 integrons carried by broad-host-range plasmids, including IncA/C, IncL/M, IncHI2 and IncN plasmids in Enterobacteriaceae^5,9,10^. In China, the *bla*_IMP_ genes have been reported in *Klebsiella* spp. belonging to different high-risk sequence types (ST) ST11, ST15 and ST307^11,12^. Among these, IMP-4 is the most common variant in IMP-producing *Klebsiella* spp. (IMPKsp) frequently located on IncN plasmids^9^. However, detailed information on the plasmids associated with other IMP variants is currently insufficient because *Klebsiella* spp. producing IMP enzymes have been rare in China. Besides, IMP enzymes often co-exist with the other carbapenemases and ESBLs in *Klebsiella* spp.^13^, including SFO-1, a rarely reported ESBL. It was firstly identified in a *Enterobacter cloacae* strain from Japan in 1999^14^, and can hydrolyze most β-lactams except carbapenems and cephamycins.

In the present study, we have performed a retrospective and descriptive study of resistance phenotypes and genomic epidemiological analysis of 61 clinical IMPKsp isolates from thirteen provinces in China from 2016-2021. We find that multiple clones, genetic environments and plasmid types are involved in disseminating *bla*_IMP_ genes among *Klebsiella* spp. These findings provide an improved understanding of the dissemination characteristics of IMPKsp strains.

## Methods

### Bacterial Isolates

A total of 61 *bla*_IMP_-positive *Klebsiella* spp. isolates were collected from thirteen provinces in China during 2016-2021. Both matrix-assisted laser desorption/ionization time of flight mass spectrometry (MALDI-TOF MS) (Bruker Daltonik GmbH, Bremen, Germany) and 16 S rRNA gene-based sequencing were applied for bacterial identification. Ethical permission for this study was approved by the Ethics Committee from The Second Affiliated Hospital of Zhejiang University, School of Medicine (2020-319).

### Antimicrobial Susceptibility Testing

The antibiotic susceptibility of all *bla*_IMP_-positive *Klebsiella* spp. isolates to common clinically used antibiotics listed in Table 1 was determined by broth microdilution method. The susceptibility results were interpreted in accordance with the Clinical and Laboratory Standards Institute (CLSI) guideline except tigecycline. The MIC of tigecycline was interpreted following US Food and Drug Administration (FDA) clinical breakpoints. *Escherichia coli* ATCC 25922 was used as a quality control strain for the antimicrobial susceptibility testing.

**Table 1:** Susceptibility of 61 IMPKsp strains to commonly used antibiotics

Antibiotics	MIC_50_(μg/mL)	MIC_90_(μg/mL)	Range(μg/mL)	R%	I%	S%
**Imipenem**	4	64	≤1– > 128	60.7%	16.4%	23.0%
Meropenem	8	128	2– > 128	96.7%	3.3%	0.0%
Ertapenem	8	>128	1- > 128	90.2%	9.8%	0.0%
Cefmetazole	>128	>128	≤2– > 128	96.7%	0.0%	3.3%
Ceftazidime	>128	>128	32- > 128	98.4%	1.6%	0.0%
Cefotaxime	>128	>128	16– > 128	100.0%	0.0%	0.0%
Piperacillin/Tazobactam	16/4	>256/4	≤8/4- > 256/4	39.3%	8.2%	52.5%
Cefoperazone/Sulbactam	128/64	>256/128	64/32- > 256/128	100.0%	0.0%	0.0%
Ceftazidime/Avibactam	64/4	>64/4	≤16/4- > 64/4	100.0%	-	0.0%
Cefepime	>64	>64	8– > 64	98.4%	-	0.0%
Colistin	≤0.5	1	≤0.5- > 8	3.3%	96.7%	-
Tigecycline	0.5	1	≤0.25-2	0.0%	0.0%	100.0%
Ciprofloxacin	≤0.25	>32	≤0.25- > 32	45.9%	0.0%	54.1%
Amikacin	≤4	>128	≤4– > 128	19.7%	0.0%	80.3%
Aztreonam	32	>128	≤4– > 128	77.0%	3.3%	19.7%

*S* susceptible, *I* intermediate-resistant, *R* resistant.

### Conjugation assay

In vitro conjugation experiments were performed using filter mating method. Rifampin-resistant *E. coli* EC600 and sodium azide-resistant *E. coli* J53 were used as the recipient strains, respectively. Transconjugants were selected on LB agar plates containing 0.5 μg/mL meropenem and 600 μg/mL rifampin and LB agar plates containing 0.5 μg/mL meropenem and 300 μg/mL sodium azide. The presence of the *bla*_IMP_ gene was confirmed by PCR and Sanger sequencing^15^.

### Whole genome sequencing and bioinformatics analysis

Genomic DNA of all *bla*_IMP_ positive *Klebsiella* spp. isolates was extracted using a HiPure Bacterial DNA Kit (Magen, China) and sequenced using the HiSeq platform (Illumina, San Diego, CA) with a 2 × 150 bp paired-end sequencing strategy^16^. Assembly was performed using SPAdes Genome Assembler version 3.11.1^17^. Location of the *bla*_IMP_ gene was identified by aligning the contigs carrying *bla*_IMP_ with complete genome sequences in the NCBI database. The genomic DNA of 13 representative *Klebsiella* spp. isolates with different resistance phenotypes and genetic traits was also sequenced using Oxford Nanopore Technologies MinION platform. The complete genome sequences of these 13 strains were obtained by hybrid assembly of Illumina and nanopore sequencing reads using Unicycler v 0.4.4^18^. The draft genomes of the remaining strains sequenced using Illumina only were aligned to the reference plasmids to deduce the plasmid types and structures. The assembled genome sequences were annotated with RAST server^19^. Besides, Kleborate v2.0.4 was used to identify the MLSTs, serotyping of KL types, antimicrobial resistance genes and virulence genes^20^. Plasmid types were identified using PlasmidFinder version 2.1^21^. Insertion sequences (ISs) were identified using ISfinder database. The generation of plasmid map was performed with BRIG v0.95. The analysis of the genetic structure of different *bla*_IMP_ genes was performed with Easyfig 2.1. Coregenome alignment and single-nucleotide polymorphism (SNP) calling were performed with The Harvest suite, and a core genome phylogenetic tree was constructed using Parsnp^22^. The phylogenetic tree was visualized and edited using iTOL version 4^23^.

### Reporting summary

Further information on research design is available in the Nature Portfolio Reporting Summary linked to this article.

## Results

### Overview of the *bla*_IMP_-positive *Klebsiella* spp. isolates

**Fig. 1:**
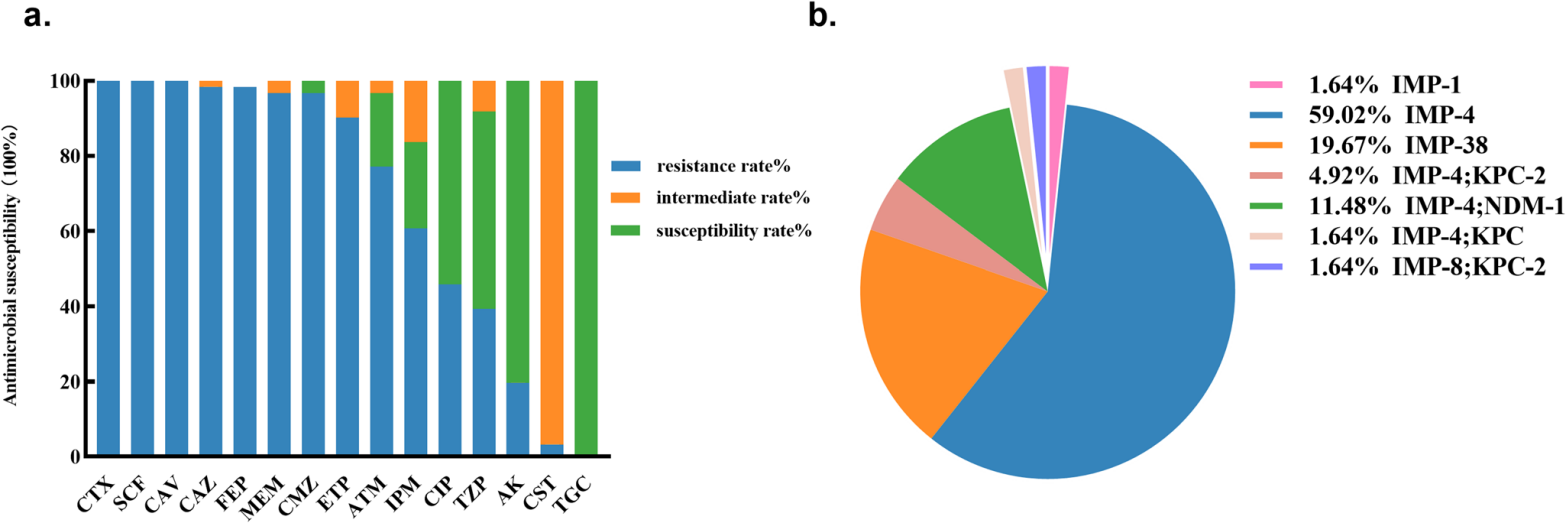
The distribution of MIC values and carbapenemases among 61 IMPKsp strains. **a** The antimicrobial susceptibility results of strains. CTX: cefotaxime; SCF: cefoperazone/sulbactam; CAV: ceftazidime/avibactam; CAZ: ceftazidime; FEP: cefepime; MEM: meropenem; CMZ: cefmetazole; ETP: ertapenem; ATM: aztreonam; IPM: imipenem; CIP: ciprofloxacin; TZP: piperacillin/tazobactam; AK: amikacin; CST: colistin; TGC: tigecycline. **b** The distribution of carbapenemases among 61 IMPKsp strains.

A total of 61 *bla*_IMP_-positive *Klebsiella* spp. strains, including 50 *K. pneumoniae*, 6 *K. variicola* subsp. *variicola*, 4 *K.quasipneumoniae* subsp. *quasipneumoniae* and 1 *K. quasipneumoniae* subsp. *similipneumoniae* were collected across China from 2016-2021. The antimicrobial susceptibility results revealed that all isolates exhibited multidrug resistance phenotypes, with >96.7% of the strains being resistant to meropenem, ceftazidime, cefotaxime, cefmetazole, cefepime, cefoperazone/sulbactam, and ceftazidime/avibactam. Besides, they manifested low to moderate resistance (19.7% to 45.9%) to amikacin, ciprofloxacin, and piperacillin/tazobactam. Only a small number of strains were resistant to colistin (3.3%), and no isolates exhibited resistance to tigecycline (Table 1, Fig. 1a).

### Antibiotic resistance genes in IMP-producing *Klebsiella* spp

In this study, *bla*_IMP_ genes were the only carbapenemase genes detected in 80.3% of these strains (49/61), with *bla*_IMP-4_ (*n* = 36) being the predominant isoform, followed by *bla*_IMP-38_ (*n* = 12) and *bla*_IMP-1_ (*n* = 1). The remaining 12 strains produced both IMP and other carbapenemases, including IMP-4 and NDM-1(*n* = 7), IMP-4 and KPC-2 (*n* = 3), IMP-4 and KPC (*n* = 1), IMP-8 and KPC-2 (*n* = 1) (Fig. 1b). The second most prevalent isoform IMP-38 differed from IMP-4 by one amino acid substitution (Ser214Gly). The IMP-8 carbapenemase derived from IMP-2 and had two amino acid substitutions (Arg214Ala and Val206Gly), compared with IMP-2.

**Fig. 2:**
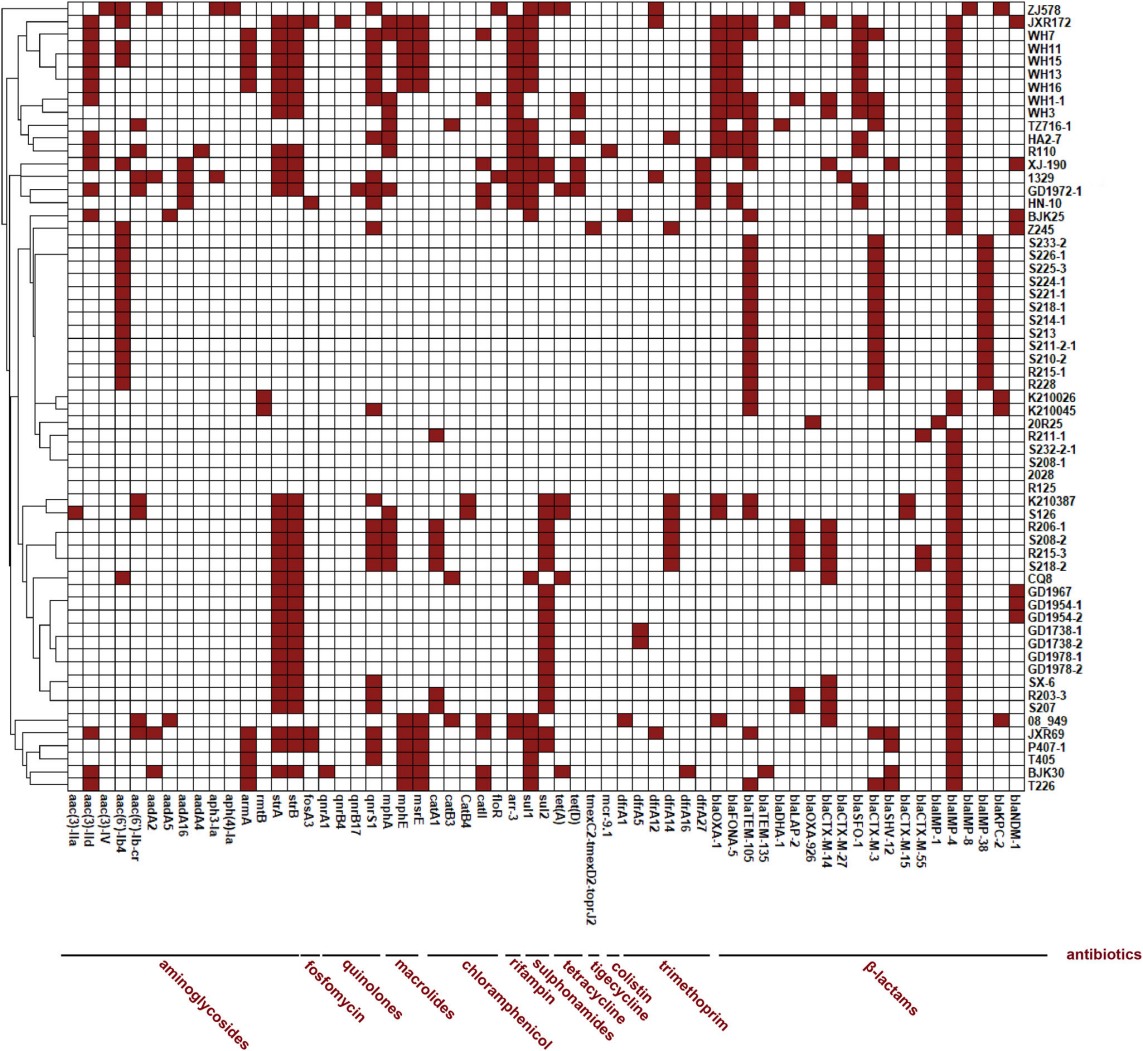
Heatmap of antibiotic resistance genes detected in 61 IMPKsp strains. The vertical and horizontal axes represent the strain numbers and antibiotic resistance genes, respectively. Red and white boxes represent the presence and absence of the antibiotic resistance genes in the strains, respectively. Names of antibiotics were displayed below the dashed lines. The genes covered by the line segment from the start point to end point could mediate resistance to labeled antibiotics.

In addition to carbapenemase genes, 57 of the 61 *bla*_IMP_-positive *Klebsiella* spp. strains also carried 1–18 other acquired antibiotic resistance genes, mainly including aminoglycoside resistance genes (*strA*, *n* = 32; *strB*, *n* = 32; *aac(6’)-Ib4*, *n* = 19; *aac(3)-IId*, *n* = 15), fluoroquinolone resistance genes (*qnrS1*, *n* = 24), sulfonamide resistance genes (*sul1*, *n* = 22; *sul2*, *n* = 21), and genes encoding extended spectrum beta lactamases (ESBLs) (*bla*_TEM-105_, *n* = 27; *bla*_CTX-M-3_, *n* = 18; *bla*_CTX-M-14_, *n* = 13; *bla*_SFO-1_, *n* = 12) (Fig. 2). Only one strain, Z245, carried a recently reported plasmid-borne multidrug resistance gene cluster, *tmexCD2*-*toprJ2*, and the minimum inhibitory concentrations (MICs) value of tigecycline for Z245 was 2 µg/mL. R110 was the unique *mcr-9.1*-positive strain but was susceptible to colistin.

### Diversity of MLSTs and phylogenetic analysis of IMP-producing *Klebsiella* spp

**Fig. 3:**
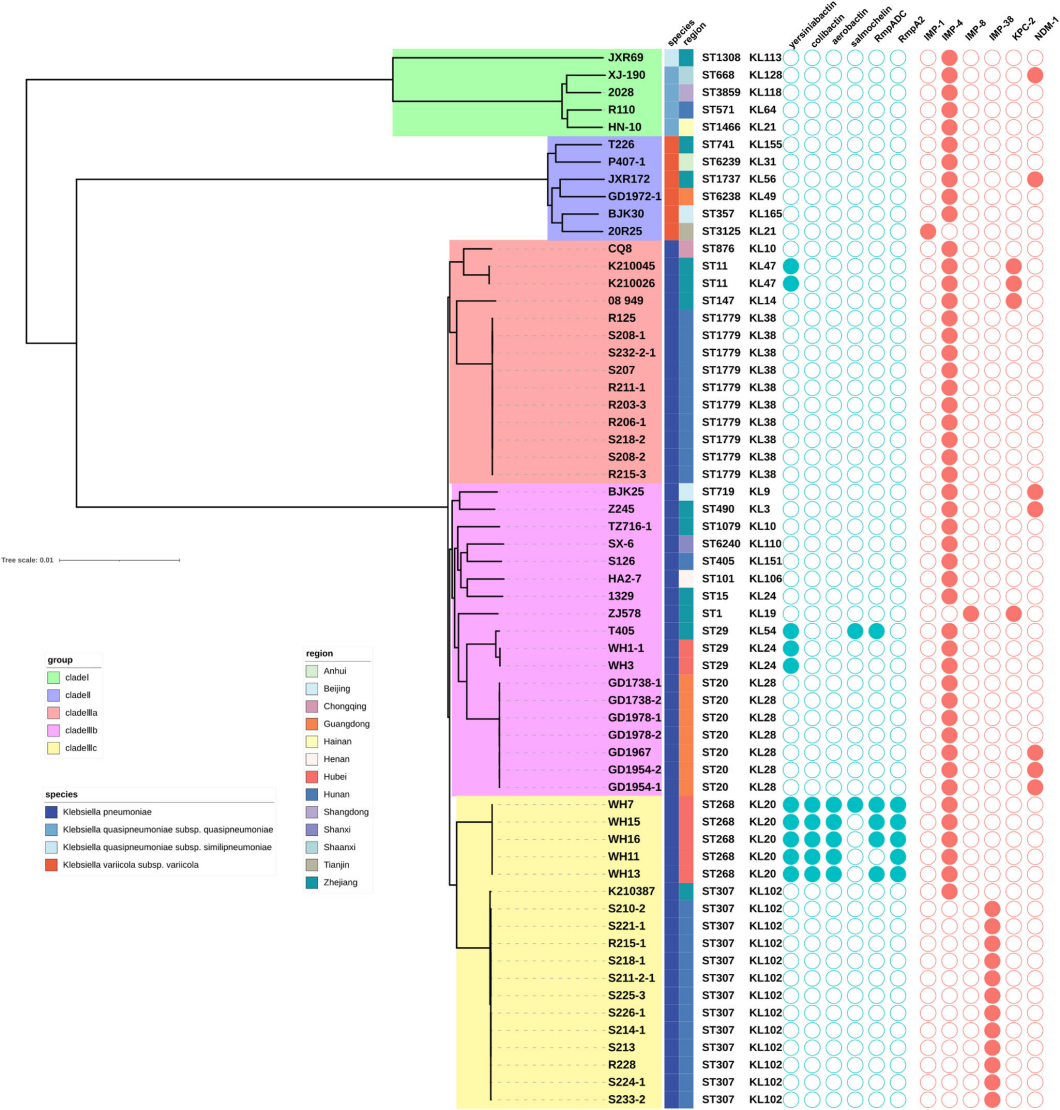
The phylogenetic tree of 61 clinical IMPKsp strains. The species, region MLST and KL serotype information are shown in order. Virulence factors and carbapenemases are indicated by circles. The solid and hollow graphics represent the presence and absence of virulence factors and carbapenemases.

The 61 IMPKsp strains belonged to 27 different STs. The most prevalent ST was ST307 (13/61, 21.3%), followed by ST1779 (10/61, 16.4%), ST20 (7/61, 11.5%) and ST268 (5/61, 8.2%). ST307, ST1779, ST20 and ST268 correspond to capsular locus (KL) 102 types, KL38, KL28 and KL20, respectively. KL20-ST268 *K. pneumoniae*, often carried virulence factors and was reported to be hypervirulence-associated^24–26^. Similarly, KL20-ST268 *K. pneumoniae* strains in this study possessed siderophore biosynthetic clusters encoding yersiniabactin, colibactin, aerobactin and/or salmochelin as well as *rmpADC* or *rmpA2* genes encoding regulators of the mucoid phenotype. Besides, KL54-ST29 strains encoded yersiniabactin, salmochelin and RmpADC. KL24-ST29 and ST11-KL47 strains produced yersiniabactin. The remaining IMPKsp strains did not harbor the aforementioned virulence factors. ST11 *K. pneumoniae*, which was the epidemic clone in China, was only detected in 3.3% (*n* = 2) of the IMPKsp strains in this study. The two ST11 *K. pneumoniae* produced both IMP-4 and KPC-2 carbapenemases. In addition, three novel ST types, including ST6238, ST6239 and ST6240, were identified in the present study. The epidemic clones exhibited to be associated with the regions. For instance, ST307 IMPKsp strains were mainly distributed in Hunan province and were primary vectors of the *bla*_IMP-38_ gene. ST1779, ST20 and ST268 IMPKsp strains were only detected in specific provinces: Hunan, Guangdong and Hubei, respectively, suggesting the clone dissemination of IMPKsp strains within these regions **(**Fig. 3).

The phylogenetic relationship analysis based on SNPs of core genomes showed that 61 IMPKsp isolates were clustered into three major clades which were associated with their species. Clade I consisted of *K. quasipneumoniae* subsp. *quasipneumoniae* and *K. quasipneumoniae* subsp. *similipneumoniae* strains (5/61, 8.2%); clade II consisted of *K. variicola* subsp. *variicola* strains (6/61, 9.8%); clade III consisted of *K. pneumoniae* (50/61, 82.0%). A total of 11 IMPKsp strains from clade I and II were sporadic and isolated from nine provinces. Clade III was further divided into three subgroups (IIIa, IIIb and IIIc), with ST1779 IMPKsp strains (10/50, 20.0%) being the major component of clade IIIa, ST20 (7/50, 14.0%) and ST29 (3/50, 6.0%) IMPKsp strains being the major component of clade IIIb, and ST307 (13/50, 26.0%) and ST268 (5/50, 10%) being the major component of clade IIIc, further suggesting a phenomenon of clone dissemination of IMPKsp strains **(**Fig. 3).

### Characteristics of *bla*_IMP_-carrying plasmids and genetic environments of *bla*_IMP_

**Fig. 4:**
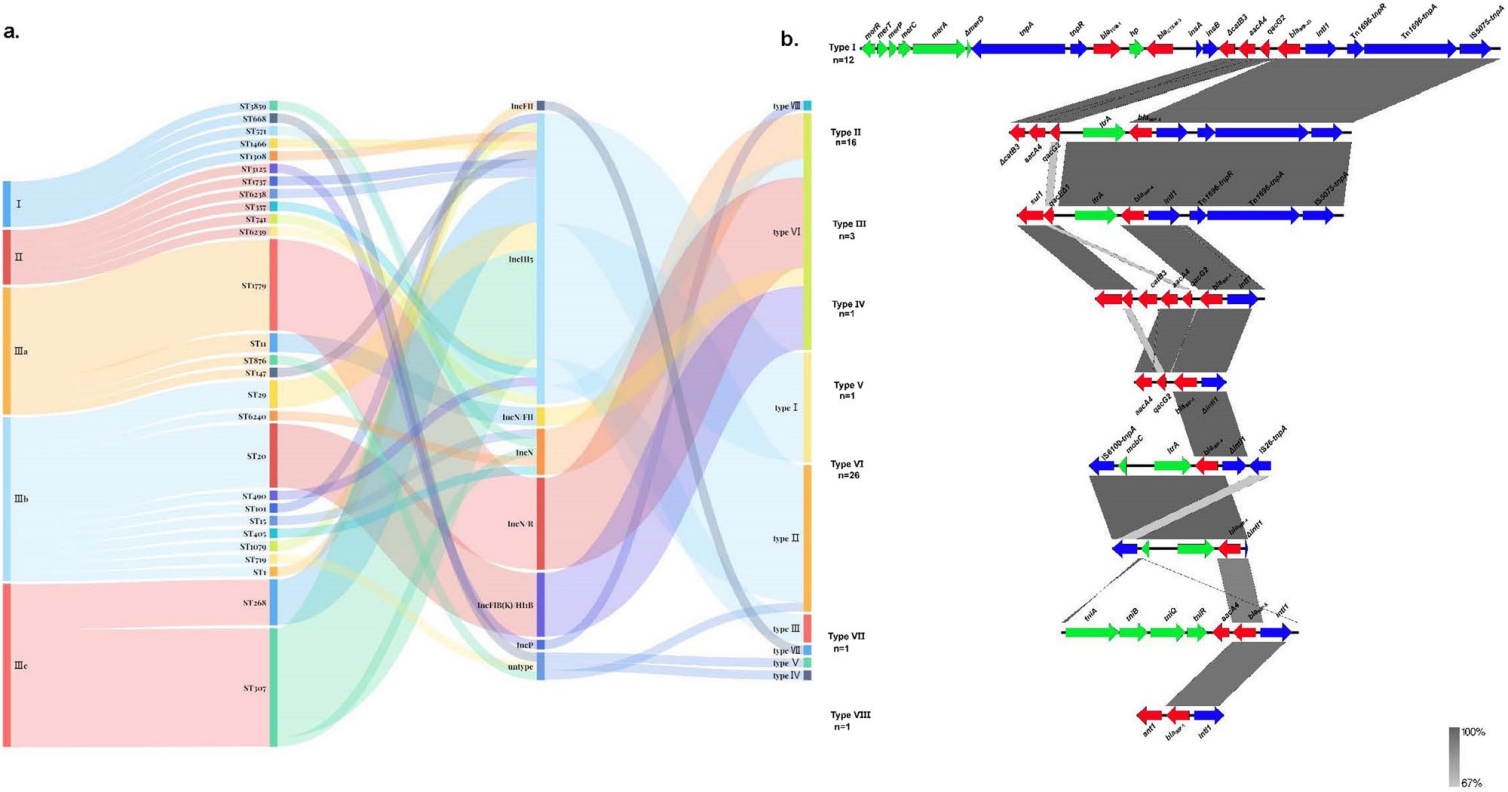
The molecular and epidemiological characteristics of clinical IMPKsp strains and genetic environment characteristics of *bla*_IMP_ genes. **a** Sankey diagram consisted of the phylogroups, MLST types, Inc types, and the genetic environment of *bla*_IMP_ genes in sequence. The width of the line is proportional to the number of isolates. **b** The alignment of genetic environment structures of *bla*_IMP_ genes among IMPKsp strains. Homologous regions are shaded in gray.

Plasmid replicon typing revealed that 58 *bla*_IMP_-carrying plasmids belonged to seven Inc types and 3 IMPKsp strains carried non-typable *bla*_IMP_-encoding plasmids. The IncHI5 (32/61, 52.5%) and IncN-IncR (10/61, 16.4%) were the predominant plasmid types, followed by IncFIB(K)-IncHI1B (7/61, 11.5%), IncN (5/61, 8.2%), IncN-IncFII (2/61, 3.3%), IncFII (1/61, 1.6%) and IncP (1/61, 1.6%) (Fig. 4a**)**. The conjugation experiments were conducted on all thirteen representative strains sequenced by Oxford nanopore sequencing technology. The results revealed that, with the exception of IncFIB(K)-IncHI1B, each type of *bla*_IMP_-carrying plasmids from six representative strains could be transferred to the recipient *E. coli* EC600 at discrepant conjugation frequencies (1.5 × 10^−6^−3.2 × 10^−3^). The IncN-IncR plasmid pIMP-R215-3 had the highest conjugation efficiency (3.2 × 10^−3^), but the IncHI5 plasmid pIMP-T405 exhibited the lowest conjugation efficiency. And the conjugation experiments were failed in the remaining seven representative isolates encoded IncHI5 *bla*_IMP_-positive plasmids, using EC600 or J53 as the recipient.

Sequence alignment suggested that thirteen *bla*_IMP_-encoding plasmids aligned well to pA708-1 from other report^27^ (Supplementary Fig. 1a); eleven *bla*_IMP_-encoding plasmids aligned well to pIMP-S225-3 (Supplementary Fig. 1b); nine *bla*_IMP_-encoding plasmids aligned well to pIMP-R215-3 (Supplementary Fig. 1c); six *bla*_IMP_-encoding plasmids aligned well to pIMP-GD1954-1 (Supplementary Fig. 1d); four *bla*_IMP_-encoding plasmids aligned to pIMP-2028 (Supplementary Fig. 1e); one *bla*_IMP_-encoding plasmids aligned well to pIMP-GD1972-1 and one *bla*_IMP_-encoding plasmid aligned well to pIMP-K210045 (Supplementary Fig. 1f, g).

Plasmid pIMP-2028 isolated from strain 2028 carrying *bla*_IMP-4_ is a representative IncN plasmid, which is 50,137 bp in size and has an average GC content of 50.24%. It was highly similar to plasmid pIMP-FJ1503 from *C. freundii* (accession no. KU051710; 100% nucleotide identity at 93% query coverage) and pIMP-ECL14-57 from a clinical *E. coli* isolate (accession no. MH727565; 99.89% nucleotide identity and 94% query coverage) (Supplementary Fig. 2a). pIMP-2028 had the intact IS*Kpn19* element but lost *qnrS1*, which located upstream of IS*Kpn19* in plasmid pIMP-FJ1503 and pIMP-ECL14-57. The *bla*_IMP-4_ with a truncated *Intl1* gene upstream was embedded in class 1 integron In*823*, which was present in a transposon-like structure IS*6100*-*mobC*-*ltrA*-*bla*_IMP-4_ -Δ*IntI1*-IS*26* (Fig. 4b). The IncN-IncR type plasmid pIMP-R215-3 was 105,677 bp in size and had GC content of 52.51% and 124 open reading frames. It showed 100% identity and 40% query coverage identity with pC52_002 (accession no. CP042547), 100% identity and 42% query coverage identity with pIMP-FJ1503 (accession no. KU051710), 99.98% identity and 40% query coverage identity with pk9 (accession no. CP049891) (Supplementary Fig. 2c). The genetic environment of *bla*_IMP-4_ gene on plasmid pIMP-R215-3 was the same as that of pIMP-2028.

The plasmid pIMP-K210045 was identified as a novel hybrid plasmid simultaneously carrying *bla*_IMP-4_ and *bla*_KPC-2_. It had 189,882 bp in length, and contained 252 open reading frames with an average GC content of 52.91%. The plasmid pIMP-K210045 carried IncN and IncFII family plasmid replication initiator RepA. Comparative genomic analysis showed that the sequence of pIMP-K210045 was composed of two main parts containing *bla*_IMP-4_ and *bla*_KPC-2_ genes, respectively. The two parts came from two different types of plasmids: the *bla*_IMP-4_ region shared 100% identity and 33% query coverage identity with pIMP-FJ1503. The genetic environment surrounding the *bla*_IMP-4_ genes was IS*6100*-*mobC*-*ltrA*-*bla*_IMP-4_-Δ*IntI1*-IS*26* (Fig. 4b); the *bla*_KPC-2_ region shared 100% identity and 73% query coverage with pKPC-2-KP65 (accession no. CP044259) of *K. pneumoniae* (Supplementary Fig. 2d**)**. The formation of hybrid plasmid was presumably linked to the presence of IS*26*.

The plasmid pIMP-GD1954-1 was 263,080 bp in length with a GC content of 51.58% and showed 99.96% identity and 82% query coverage identity with a hybrid plasmid pAZS099-NDM-IMP (accession no. CP086762) carrying both *bla*_IMP-4_ and *bla*_NDM-1_ in a ST20-KL28 *K. pneumoniae* (Supplementary Fig. 2e**)**. The *bla*_IMP-4_ gene was located in the genetic environment IS*6100*-*mobC*-*ltrA*-*bla*_IMP-4_-Δ*IntI1*-IS*26* (Fig. 4b). The remnant region of pAZS099-NDM-IMP with *bla*_NDM-1_ gene was also detected in the form of a circular IncX3-type plasmid in the strain GD1954-1 and named pNDM-GD1954-1. Further analysis showed that IS*6*-like element IS*26* family transposase led to the formation of two single plasmids originating from pAZS099-NDM-IMP. It is known that IncX3-type plasmid is an easily transmissible vector. And in the filter-mating assay in this study, only transconjugants with *bla*_NDM-1_ gene was obtained and no transconjugants carrying *bla*_IMP-4_ gene was obtained.

The IncFII plasmid pIMP-ZJ578 was 45,535 bp long and had an average GC content of 49.31%, encoding 57 ORFs. This plasmid showed 99.99% nucleotide identity and 99% query coverage with p16005813B (MK036884), which was isolated from clinical *Leclercia adecarboxylata* in Ningbo, China (Supplementary Fig. 2f**)**. The *bla*_IMP-8_ was located on a class 1 integron In655 (*tniA*-*tniB*-*tniQ*-*tniR*-*aacA4*-*bla*_IMP-8_-Δ*IntI1*) (Fig. 4b), which was carried by Tn*6505* derived from Tn*1696*. The Tn*6505* of pIMP-ZJ578 differed from that of p16005813B by insertion of 45 nucleotides in *merT* gene.

The pIMP-20R25 was an IncP-1β type plasmid encoding *bla*_IMP-1_ gene and had a circularly closed 61,282 bp DNA sequence consisting of 68 ORFs, with an average GC content of 63.14%. Sequence analysis showed that the pIMP-20R25 was highly homologous to another *bla*_IMP-1_-carrying IncP-1β type plasmid pNXM63-IMP (accession no. NZ_MW150990; coverage, 88%; identity, 99.99%) found in *Morganella morganii* and plasmid pIMP4-ECL352 (accession no. CP083711; coverage, 95%; identity, 99.99%) (Supplementary Fig. 2b). The *bla*_IMP-1_ was located in a novel genetic environment of *ant1*-*bla*_IMP-1_-Δ*IntI1* (Fig. 4b), which was firstly identified to be adjacent to *ant1*, a streptomycin resistance gene. The existence of two putative transfer regions belonging to the IncP-1β plasmid backbone made it self-transmissible easily. So far, IMP-1-encoding IncP-1β plasmids were only detected in *Achromobacter xylosoxidans* and *M. morganii*^28,29^. This is the first report of *K. variicola subsp. variicola* harboring a conjugative *bla*_IMP-1_-positive IncP-1β plasmid.

The seven plasmids including pIMP-GD1972-1, pIMP-P407-1, pIMP-R110, pIMP-S225-3, pIMP-T226, pIMP-T405, and pIMP-Z245 belonged to IncHI5 incompatibility group in this study. The plasmids varied in size from about 223 kb to nearly 377 kb and the number of predicted ORFs varied from 241 to 424. The seven plasmids described above carried *bla*_IMP-4_ gene except that pIMP-S225-3 carried *bla*_IMP-38_ gene. IncHI plasmids are important vectors of genes encoding for antibiotic resistance, such as β-lactams including carbapenems, aminoglycosides, and quinolones. In this study, IncHI5 plasmid harbored more antibiotic resistance genes than other types of plasmids, leading to multi-drug resistance and facilitating survival under different antimicrobial pressures. According to the previous report, *bla*_IMP_ genes were generally located in the transposons or transposon-like structures on the antibiotic resistance islands designated ARI-A. In the present work, the *bla*_IMP-4_ gene was possessed by a class I integron within Tn*1696*-Related ARI-A with the gene cassette of IS*CR1-sul1*- *qacE*Δ1-*ltrA*-*bla*_IMP-4_ -*IntI1*-*tnpR*-*tnpA*-IS5075 on pIMP-P407-1, pIMP-T226 and pIMP-T405, consistent with that of p11219-IMP (accession no. MF344561) (Supplementary Fig. 3b-d**)**. The genetic environment of the *bla*_IMP-4_ gene was Δ*catB3*-*aacA4*-*ltrA*-*bla*_IMP-4_-*IntI1*-*tnpR*-*tnpA*-IS*5075* on pIMP-R110 and pIMP-Z245, consistent with that of pA708-1(accession no. CP026369) (Fig. 4b, Supplementary Fig. 3f,g**)**. The ARI-A islands were not found in pIMP-GD1972-1 (Supplementary Fig. 3e**)**. The *bla*_IMP-4_ gene of pIMP-GD1972-1was located in the genetic environment IS*6100*-*mobC*-*ltrA*-*bla*_IMP-4_-Δ*IntI1*-IS*26* (Fig. 4b). The pIMP-S225-3 showed 100% nucleotide identity and 82% query coverage with pA324-IMP (accession no. MF344566) (Supplementary Fig. 3a**)**. They both had the intact Tn*1696* derivatives-Tn*6382* bearing *bla*_CTX-M-3_ and *bla*_TEM-1B_ genes, and the *bla*_IMP-38_ gene was also located within Tn*6382* with the gene cassette of Δ*catB3*-*aacA4*-*qacG2*-*bla*_IMP-38_- *IntI1* (Fig. 4b).

Together, these results suggested the plasmid types and genetic environment of *bla*_IMP_ genes were diversified and there was a certain correlation between them. Most of the *bla*_IMP_ genes located on the IncHI5 plasmids were associated with the ARI-A islands. IncHI5 plasmid was the predominant plasmid type and carried complex antibiotic resistance genes. The strains carrying IncHI5 plasmids belonged to diverse ST types, indicating that they played an important role in the transmission of *bla*_IMP_ genes among *Klebsiella spp*. strains.

## Discussion

The ongoing epidemic of carbapenemase-producing *Klebsiella* spp. has emerged as a mounting menace for human health worldwide^1^. However, the prevalence and genomic charaterization of IMPKsp isolates are less studied nationwide due to that KPC, OXA-48-like and NDM enzymes are the major carbapenemases in CRKP strains in China, and they have been widely studied^4^. The prevalence of IMP in *Klebsiella* spp. is relatively low and often ignored. Large-scale studies on the “ancient” carbapenemase IMP in *K. pneumoniae* are still lacking.This descriptive work investigated molecular and epidemiological characteristics of clinical IMPKsp strains in China and revealed that the genetic background of *bla*_IMP_ genes was diverse and IncHI5 plasmid has the potential to be another important vector mediating transmission of *bla*_IMP_.

Only one IMPKsp strain was carbapenem-susceptible. A previous study reported that the transcription factor ArdK encoded by plasmids inhibited the expression of the IMP-6 metallo-β-lactamase, conferring a carbapenem-susceptible phenotype in the *bla*_IMP-6_-positive *E. coli* strain^30^. Further research is needed to explain the mechanism of carbapenem susceptibility of IMP-positive isolates.

The IMP-type MBL was first identified in *Klebsiella* spp. from Wuhan in China in 2008^31^. Since then, sporadic cases of IMPKsp infections have been frequently reported nationwide. And there have also been sporadic epidemics caused by IMPKsp strains. IMP-38-producing ST307 *K. pneumoniae* strains and IMP-4-producing ST2253 *K. pneumoniae* infected neonatal patients in the neonatal ward in Shandong and Hunan province, respectively^11,32^. This again is consistent with our findings that there was a regional spread of ST307 IMPKsp strains carrying IMP-38 carbapenemases in Hunan province. *K. pneumoniae* ST307 has emerged as an antimicrobial-resistant high-risk clone during the 1990s and has been associated with the transmission of various carbapenemases worldwide^11,33–35^. Control measures are urgently required to prevent the transmission rates of ST307 *K. pneumoniae* producing IMP carbapenemase. In this study, another epidemic clone IMP-4-producing ST1779 *K. pneumoniae* was first detected in Hunan province. Though this new ST1779 was not relevant to other well-known epidemic IMP-4 producing *K. pneumoniae* clones reported in China, this novel ST might become a high-risk clone that needed close attention. ST20 was the one of the most common *K. pneumoniae* ST type in China and was the third most frequently observed clone in the present study. ST20 *K. pneumoniae* was the common vector of IMP-4 and NDM-1 carbapenemases^36^. Recently, Jia et al. have first reported a novel hybrid plasmid coharboring IMP-4 and NDM-1 carbapenemases in ST20 *K. pneumoniae*^37^. But in our study, the *bla*_IMP-4_ and *bla*_NDM-1_ genes were located on two different plasmids in ST20 *K. pneumoniae*, indicating that this ST could also pose a potential threat to human health. These reflected the vertical transmission of *bla*_IMP_ genes. Therefore, it is necessary to monitor the spread of specific epidemic clones in specific regions to combat this gene.

In this study, plasmid spread was the main transmission mode of *bla*_IMP-4_ gene, though clonal spread, and integron spread were also observed. IncHI5 was the predominant type of *bla*_IMP_-harboring plasmid in the isolates investigated and involved a variety of ST clones in the study, suggesting the potential of horizontal transfer of IncHI5-type plasmids among *Klebsiella* spp. Though this type of plasmids has broad host range, the low transformation efficiency or non-transferability of plasmids has been observed in this study, probably due to the nonoptimal conjugation condition, which required further study. So far, *bla*_IMP-4_ and *bla*_IMP-38_ genes have been found to reside on IncHI5 plasmids in three previous studies from China^11,38^, which is consistent with our findings. A previous study has reported that another subgroup of IncHI plasmid, IncHI2 plasmid has led to the high prevalence of *bla*_IMP-4_ gene, which encoded the major carbapenemases in carbapenemase-producing Enterobacteriaceae (CPE) in Queensland^39^. Similarly, extra vigilance is required that *bla*_IMP_ gene may be maintained in the *Klebsiella* spp. population by circulating on IncHI5 plasmids or via clonal expansion in China.

Synthesizing across previous researches, the IncN plasmids were the major carrier of *bla*_IMP_ gene among *Klebsiella* spp. and play an important role in transmission of carbapenemases^9,13,40^. And in our study, the broad-host-range and conjugative IncN plasmid was detected in diverse ST types, and the fusion events between IncN and other types of plasmids including IncR and IncFII plasmids were observed, indicating a potential route for the evolution of IncN plasmids.

In addition to the variety of plasmid types, class 1 integrons linked to autonomously transferable genetic structures surrounding *bla*_IMP_ genes were also divergent, and were the vehicles for dissemination of *bla*_IMP_ genes^9^, contributing to the diversity of plasmids in this study. Though the structure IS*6100*-*mobC*-*ltrA*-*bla*_IMP-4_-Δ*IntI1*-IS*26* was highly conserved, the plasticity of genetic contexts of *bla*_IMP_ genes in IncHI5 plasmid was observed in the study. Multiple insertional elements, mainly IS*26* and IS*5075*, were involved in the genetic structure alterations of *bla*_IMP_ gene.

The *bla*_SFO-1_ gene was usually detected in *Enterobacter* spp. in China and has been reported only once in association with *bla*_IMP-4_ gene in *K. pneumoniae*^41,42^. But our study revealed that the *bla*_SFO-1_ gene is not as uncommon as previously considered. Besides, among the 12 *bla*_SFO-1_-positive IMPKsp strains, we found the coexistence of *bla*_SFO-1_ gene and *bla*_IMP-4_ on the IncHI5 plasmids in 11 strains. The 11 strains belonged to six different ST types, suggesting the potential for horizontal transfer of the *bla*_SFO-1_ gene along with the IncHI5 plasmids. Ai et al. has reported that *mcr-9*, *bla*_NDM−1_ and a rare gene *bla*_SFO−1_ were detected simultaneously on the same IncHI2-ST1 plasmid^43^. This plasmid type often led to the spread of carbapenemase-encoding genes and the evolution of complex resistance phenotypes, so did IncHI5 plasmid. The prevalence of *bla*_SFO-1_-positive strains might be underestimated, and more international surveillance of *bla*_SFO-1_-positive strains and IncHI plasmids should be adopted to prevent their dissemination. Besides, colistin and tigecyline were recognized as the last line of defense against severe infections, but the coexistence of *bla*_IMP_ genes with colistin resistance gene *mcr-9.1* or tigecycline resistance gene *tmexCD2*-*toprJ2*, was observed in this study, aggravating the problem of antibiotic resistance. Though the *mcr-9.1* did not confer resistance to colistin in this study, continued vigilance was needed to the “silent” transmission of *mcr-9.1*, especially to strains with *qseB/qseC* two-component system, which could regulate expression of *mcr-9.1* gene^44^.

There are some limitations in this descriptive work. 61 IMP-positive isolates were collected from 13 provinces rather than all provinces in China. No positive strains were detected in some provinces. Phenotypic and genomic characteristics of 61 IMP-positive isolates could represent most but not all regions of China.

In conclusion, this descriptive work elucidated the phenotypic and genotypic characteristics of plasmids containing *bla*_IMP_ genes in *Klebsiella* spp. The IMPKsp isolates exhibited high sequence diversity. The dissemination of *bla*_IMP_ genes was driven by the multiple genetic environment and plasmid types. Compared with previous studies, IncHI5-type plasmids may be developed into epidemic types in *Klebsiella* spp. in China. Class 1 integrons still play an important role in the dissemination of *bla*_IMP_ genes. The primary epidemic clones have regional differences in several provinces in China, suggesting that stringent monitoring and appropriate actions are needed.

### Supplementary information

Supplementary Information

Supplementary Data 1

Supplementary Data 2

Reporting Summary

Description of Additional Supplementary Files

## Data Availability

The genome sequences in this study were deposited in the NCBI database under the BioProject PRJNA887693. The source data underlying the graphs and charts presented in the main figures can be found in Supplementary Data 1 and 2.
