# Supplementary material for: Phenotypic and genomic characteristics of clinical IMP-producing Klebsiella spp. Isolates in China
Source: Commun Med (Lond). 2024 Feb 21;4:25. doi: 10.1038/s43856-024-00439-5 (PMC10881498; doi:10.1038/s43856-024-00439-5)
Supplement: Supplementary file 1 — Supplementary Information [file 43856_2024_439_MOESM1_ESM.pdf]

Supplementary Information

Table S1. The antimicrobial susceptibility results of IMPKp and transconjugant strains.

Strain	IPM	MEM	ETP	CMZ	CAZ	CTX	TZP	SCF	CAV	FEP	CST	TGC	CIP	AK	ATM
P407-1	2	2	1	>128	>128	64	≤8/4	64/4	>64/4	16	≤0.5	≤0.25	⩽0.25	≤4	128
R110	2	4	1	>128	128	64	64/4	128/64	64/4	16	≤0.5	1	⩽0.25	≤4	128
R125	4	8	4	128	64	64	≤8/4	64/32	>64/4	16	≤0.5	0.5	⩽0.25	≤4	≤4
R203-3	4	16	4	>128	>128	>128	32/4	256/128	>64/4	>64	≤0.5	≤0.25	4	≤4	8
R206-1	4	4	4	>128	>128	>128	≤8/4	128/64	>64/4	>64	≤0.5	≤0.25	2	8	32
R211-1	2	8	1	>128	128	>128	16/4	128/64	>64/4	>64	≤0.5	≤0.25	2	≤4	128
R215-1	≤1	8	8	128	32	>128	≤8/4	64/32	32/4	>64	≤0.5	0.5	⩽0.25	≤4	32
R215-3	4	8	4	>128	>128	>128	16/4	128/64	>64/4	>64	≤0.5	≤0.25	2	≤4	128
R228	≤1	8	8	128	32	>128	≤8/4	64/32	32/4	>64	≤0.5	≤0.25	⩽0.25	≤4	32
S126	2	8	4	>128	>128	>128	32/4	128/64	>64/4	>64	2	1	8	≤4	>128
S207	4	8	1	>128	128	>128	16/4	128/64	>64/4	64	1	≤0.25	2	≤4	≤4
S208-1	2	4	4	128	64	64	≤8/4	64/32	>64/4	16	1	0.5	⩽0.25	≤4	≤4
S208-2	2	8	4	>128	>128	>128	≤8/4	128/64	>64/4	64	≤0.5	≤0.25	2	≤4	16
S210-2	≤1	8	4	>128	32	>128	≤8/4	64/32	32/4	64	1	≤0.25	⩽0.25	≤4	32
S211-2-1	≤1	16	8	>128	32	>128	≤8/4	64/32	32/4	>64	1	≤0.25	⩽0.25	≤4	32
S213	≤1	16	8	>128	32	>128	≤8/4	64/32	32/4	>64	≤0.5	≤0.25	⩽0.25	≤4	32
S214-1	≤1	16	4	128	32	>128	≤8/4	64/32	32/4	>64	≤0.5	≤0.25	⩽0.25	≤4	32
S218-1	≤1	16	8	128	32	>128	≤8/4	128/64	64/4	>64	≤0.5	0.5	⩽0.25	≤4	32
S218-2	≤1	8	1	>128	>128	>128	16/4	128/64	>64/4	>64	≤0.5	≤0.25	2	≤4	128
S221-1	≤1	16	4	128	32	>128	≤8/4	64/32	32/4	>64	≤0.5	≤0.25	⩽0.25	≤4	32
S224-1	≤1	16	8	128	32	>128	≤8/4	64/32	32/4	>64	≤0.5	≤0.25	⩽0.25	≤4	64

S225-3	≤1	16	8	>128	32	>128	≤8/4	128/64	64/4	>64	≤0.5	≤0.25	⩽0.25	≤4	64
S226-1	≤1	16	4	>128	32	>128	≤8/4	64/32	32/4	>64	≤0.5	≤0.25	⩽0.25	≤4	32
S232-2-1	4	8	4	>128	128	128	≤8/4	64/32	>64/4	16	≤0.5	0.5	⩽0.25	≤4	≤4
S233-2	≤1	16	8	128	32	>128	≤8/4	64/32	32/4	>64	≤0.5	≤0.25	⩽0.25	≤4	32
2028	4	8	8	>128	>128	64	16/4	64/32	>64/4	32	≤0.5	1	⩽0.25	≤4	≤4
20R25	2	4	4	128	128	64	≤8/4	64/32	16/4	16	≤0.5	2	⩽0.25	≤4	≤4
GD1738-1	4	8	8	>128	>128	128	128/4	256/128	32/4	64	≤0.5	0.5	⩽0.25	≤4	≤4
GD1738-2	8	8	32	>128	>128	128	256/4	256/128	64/4	>64	≤0.5	0.5	2	≤4	>128
GD1954-1	8	16	16	>128	>128	128	256/4	>256/128	>64/4	32	1	0.5	⩽0.25	8	>128
GD1954-2	8	16	16	>128	>128	128	256/4	256/128	>64/4	32	2	0.5	⩽0.25	≤4	>128
GD1967	8	16	16	>128	>128	128	>256/4	>256/128	>64/4	64	≤0.5	0.5	⩽0.25	≤4	>128
GD1972-1	4	4	8	>128	>128	128	16/4	>256/128	>64/4	64	≤0.5	1	4	≤4	32
GD1978-1	8	8	16	>128	>128	128	32/4	256/128	>64/4	64	≤0.5	1	⩽0.25	≤4	≤4
GD1978-2	8	8	16	>128	>128	128	16/4	256/128	>64/4	64	≤0.5	0.5	⩽0.25	≤4	≤4
T226	2	8	8	>128	>128	>128	16/4	128/64	64/4	>64	≤0.5	1	⩽0.25	≤4	>128
T405	≤1	4	4	>128	>128	128	>256/4	128/64	32/4	64	1	2	4	128	>128
TZ716-1	8	8	8	>128	>128	>128	256/4	>256/128	>64/4	>64	≤0.5	1	⩽0.25	≤4	64
Z245	8	16	8	>128	>128	128	>256/4	>256/128	>64/4	32	≤0.5	2	2	≤4	≤4
ZJ578	>128	>128	>128	>128	>128	>128	>256/4	>256/128	>64/4	>64	≤0.5	1	>32	≤4	>128
K210026	128	>128	>128	>128	>128	>128	>256/4	>256/128	4/4	>64	4	2	>32	>128	128
K210045	64	128	>128	>128	>128	>128	>256/4	>256/128	8/4	>64	≤0.5	1	>32	>128	>128
K210387	4	4	>128	>128	>128	>128	64/4	128/64	>64/4	32	≤0.5	0.5	>32	≤4	32
BJK25	64	128	>128	>128	64	>128	>256/4	>256/128	≤0.5/4	>64	≤0.5	1	32	>128	>128
BJK30	4	4	4	>128	128	128	128/4	256/128	64/4	32	>8	1	⩽0.25	≤4	≤4
CQ8	4	4	4	>128	>128	>128	16/4	128/64	>64/4	32	≤0.5	0.5	⩽0.25	≤4	32
HA2-7	2	4	1	128	128	>128	≤8/4	256/128	8/4	32	≤0.5	0.5	⩽0.25	≤4	16

HN-10	2	4	4	>128	>128	>128	16/4	>256/128	64/4	32	≤0.5	1	⩽0.25	≤4	128
JXR172	16	32	128	>128	>128	16	>256/4	64/32	8/4	>64	≤0.5	2	>32	≤4	16
JXR69	4	2	16	>128	>128	>128	>256/4	128/64	≤0.5/4	>64	≤0.5	1	>32	>128	>128
SX-6	4	4	4	>128	>128	>128	16/4	128/64	>64/4	32	≤0.5	0.5	⩽0.25	≤4	≤4
WH11	8	16	16	8	>128	64	256/4	64/32	≤0.5/4	8	≤0.5	1	32	>128	>128
WH1-1	64	128	>128	>128	>128	>128	>256/4	256/128	≤0.5/4	>64	≤0.5	1	>32	>128	>128
WH13	128	128	>128	>128	>128	>128	>256/4	>256/128	4/4	>64	≤0.5	1	>32	>128	>128
WH15	64	128	>128	>128	>128	>128	>256/4	256/128	≤0.5/4	>64	≤0.5	1	>32	>128	>128
WH16	128	128	>128	>128	64	>128	>256/4	>256/128	1/4	>64	≤0.5	2	>32	>128	>128
WH3	128	>128	>128	>128	128	>128	>256/4	>256/128	2/4	>64	≤0.5	1	>32	>128	>128
WH7	8	32	8	≤2	>128	32	>256/4	256/128	≤0.5/4	64	≤0.5	1	32	>128	>128
XJ-190	16	32	32	>128	>128	>128	>256/4	>256/128	>64/4	>64	≤0.5	0.5	⩽0.25	≤4	>128
1329	4	8	16	>128	128	>128	≤8/4	64/32	>64/4	32	≤0.5	1	>32	≤4	8
08_949	8	16	16	>128	>128	>128	>256/4	256/128	>64/4	64	0.5	≤0.25	8	≦4	>128
R215-3-TC	2	2	1	>128	>128	>128	16/4	128/64	>64/4	64	≤0.5	≤0.25	2	≤4	128
ZJ578-TC	16	8	16	64	128	64	>256/4	256/128	64/4	32	≤0.5	0.5	2	≤4	>128
2028-TC	2	2	1	>128	>128	64	16/4	64/32	>64/4	32	≤0.5	0.5	≤0.25	≤4	≤4
S218-2-TC	≤1	2	1	>128	>128	128	16/4	128/64	>64/4	32	≤0.5	⩽0.25	⩽0.25	≤4	≤4
20R25-TC	2	4	4	128	128	64	≤8/4	64/32	>64/4	16	≤0.5	⩽0.25	⩽0.25	≤4	≤4
K210045-TC	16	16	32	64	64	32	>256/4	256/128	2/4	32	≤0.5	0.5	2	>128	>128
T405-TC	≤1	2	1	>128	>128	64	≤8/4	256/128	>64/4	16	≤0.5	0.5	≤0.25	128	>128

IPM: imipenem; MEM: meropenem; ETP: ertapenem; CMZ: cefmetazole; CAZ: ceftazidime; CTX: cefotaxime; TZP: piperacillin/tazobactam; SCF: cefoperazone/sulbactam; CAV: ceftazidime/avibactam; FEP: cefepime; CST: colistin; TGC: tigecycline; CIP: ciprofloxacin; AK: amikacin; ATM: aztreonam.

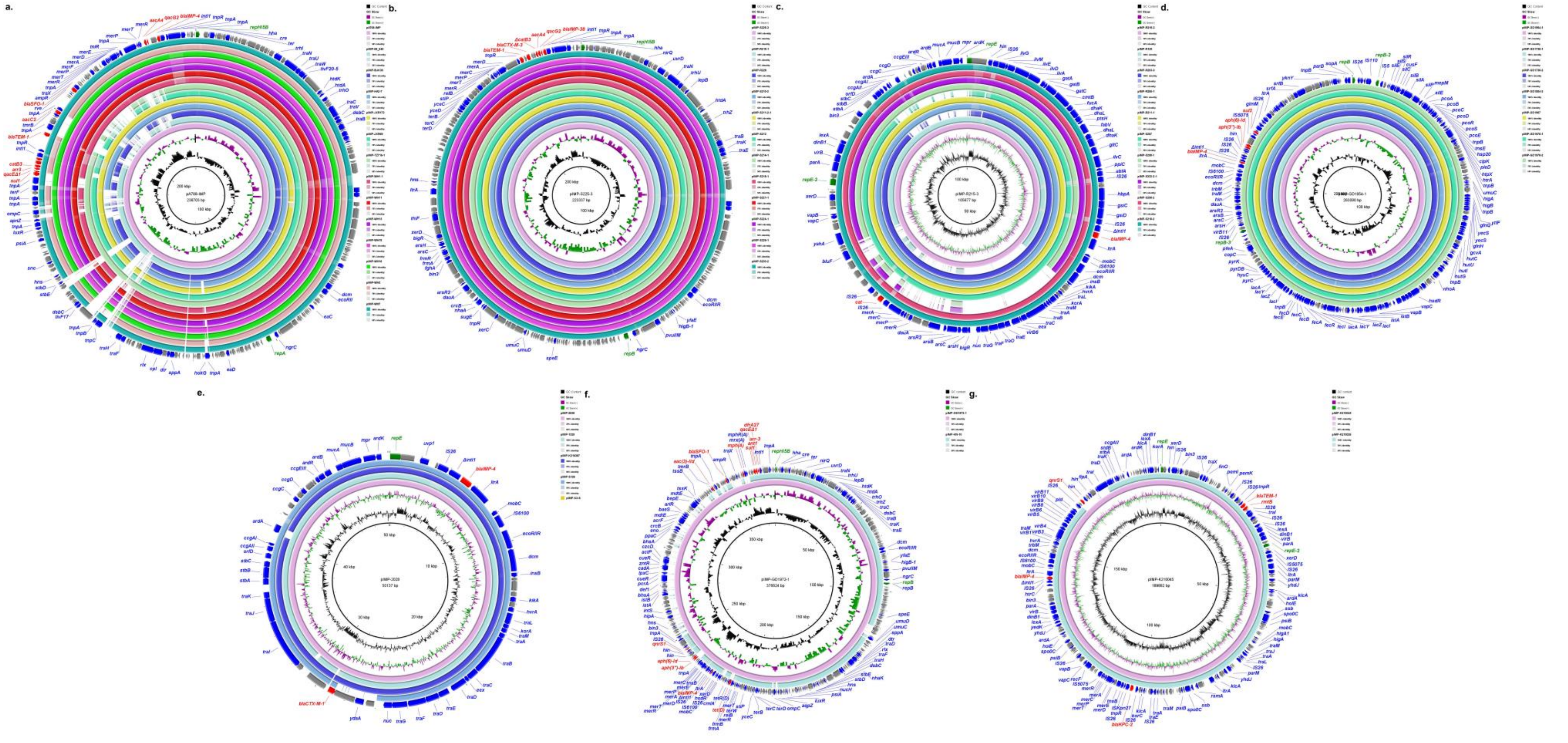

Supplementary Figure 1. Alignment of *bla*$_{\text{IMP}}$-encoding plasmids in strains sequenced by Illumina technology with complete plasmids. (a)

Circular comparison between plasmid pA708-IMP and other similar plasmids. (b) Circular comparison between plasmid pIMP-S225-3 and other similar plasmids. (c) Circular comparison between plasmids pIMP-R215-3 and similar plasmids. (d) Circular comparison between plasmids pIMP-pIMP-GD1954-1 and similar plasmids. (e) Circular comparison between plasmids pIMP-2028 and similar plasmids. (f) Circular comparison between plasmids pIMP-GD1972-1 and similar plasmids. (g) Circular comparison between plasmids pIMP-K210045 and similar plasmids. The different colors represented different plasmids in each subfigure.

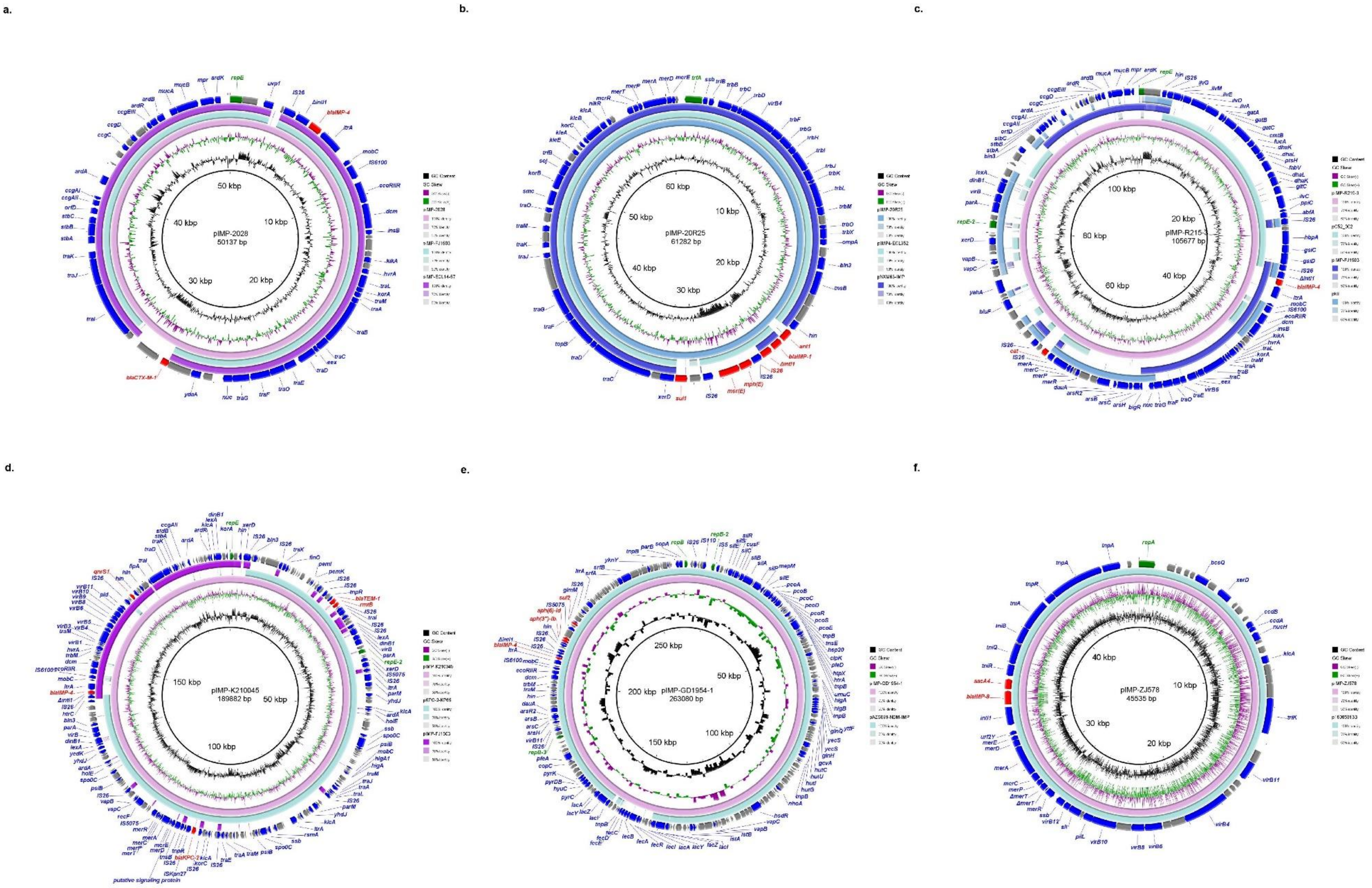
a.
pIMP-2028
50137 bp
b.
pIMP-20R25
61282 bp
c.
pIMP-R215-3
105677 bp
d.
pIMP-K210045
189882 bp
e.
pIMP-GD1954-1
263080 bp
f.
pIMP-ZJ578
45535 bp

Supplementary Figure 2. Alignment of *bla*$_{IMP}$-encoding plasmids with homologous plasmids. (a) Circular comparison between plasmid pIMP-2028 and other similar plasmids. (b) Circular comparison between plasmid pIMP-20R25 and other similar plasmids. (c) Circular comparison between plasmids pIMP-R215-3 and similar plasmids. (d) Circular comparison between plasmids pIMP-K210045 and similar plasmids. (e) Circular comparison between plasmids pIMP-GD1954-1 and similar plasmids. (f) Circular comparison between plasmids pIMP-ZJ578 and similar plasmids. The different colors represented different plasmids in each subfigure.

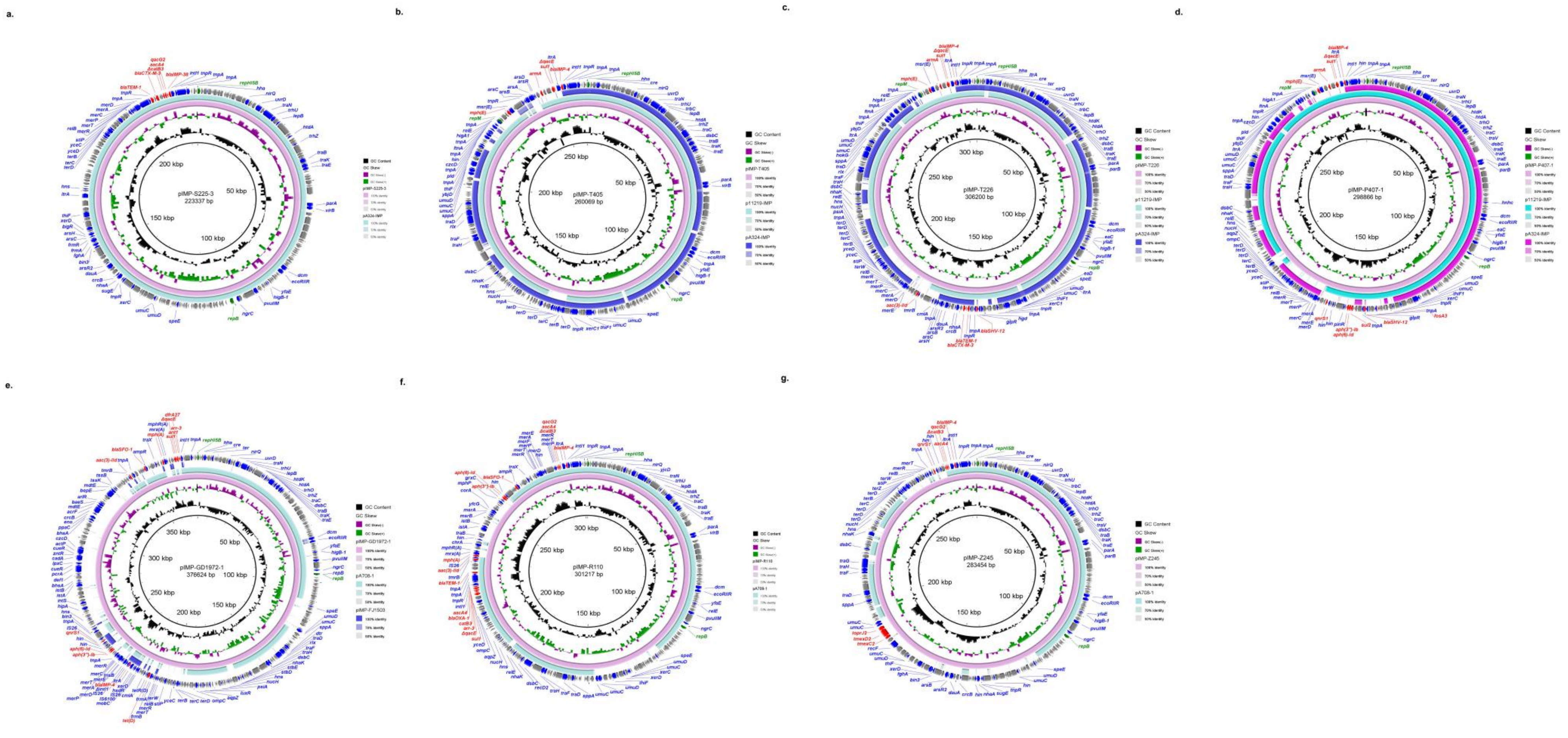

Supplementary Figure 3. Alignment of *bla*$_{\mathrm{IMP}}$-encoding IncHI5 plasmids with homologous plasmids. (a) Circular comparison between plasmid pIMP-S225-3 and other similar plasmids. (b) Circular comparison between plasmid pIMP-T405 and other similar plasmids. (c) Circular comparison between plasmids pIMP-T226 and similar plasmids. (d) Circular comparison between plasmids pIMP-P407-1 and similar plasmids. (e) Circular comparison between plasmids pIMP-GD1972-1 and similar plasmids. (f) Circular comparison between plasmids pIMP-R110 and similar plasmids. (g) Circular comparison between plasmids pIMP-Z245 and similar plasmids. The different colors represented different plasmids in each subfigure.
