# Supplementary material for: Phenotypic and genomic characteristics of clinical IMP-producing Klebsiella spp. Isolates in China
Source: Commun Med (Lond). 2024 Feb 21;4:25. doi: 10.1038/s43856-024-00439-5 (PMC10881498; doi:10.1038/s43856-024-00439-5)
Supplement: Supplementary file 5 — Description of Additional Supplementary Files [file 43856_2024_439_MOESM5_ESM.pdf]

Description of Additional Supplementary Files

File Name: Supplementary Data 1
Description: The susceptibility of 61 IMPKsp strains to commonly used antibiotics

File Name: Supplementary Data 2
Description: The antibiotic resistance genes detected in 61 IMPKsp strains
